# Prevalence of atrial fibrillation in a regional Victoria setting, findings from the crossroads studies (2001–2003 and 2016–2018)

**DOI:** 10.1111/ajr.12914

**Published:** 2022-08-08

**Authors:** Kristen Glenister, Leslie Bolitho, Lisa Bourke, David Simmons

**Affiliations:** ^1^ Department of Rural Health University of Melbourne Wangaratta Vic. Australia; ^2^ Wangaratta Cardiology & Respiratory Centre Wangaratta Vic. Australia; ^3^ Department of Rural Health University of Melbourne Shepparton Vic. Australia; ^4^ School of Medicine Western Sydney University Campbelltown NSW Australia

**Keywords:** AliveCor®, arrhythmia, atrial fibrillation, rural, screening

## Abstract

**Objective:**

To estimate the prevalence of atrial fibrillation (AF) in regional Victoria at two time points (2001–2003 and 2016–2018), and to assess the use of electrocardiogram rhythm strips in a rural, community‐based study for AF investigation.

**Design:**

Repeated cross‐sectional design involving survey of residents of randomly selected households and a clinic. Predictors of AF were assessed using Firth penalised logistic regression, as appropriate for rare events.

**Setting:**

Goulburn Valley, Victoria.

**Participants:**

Household residents aged ≥16 years. Non‐pregnant participants aged 18+ were eligible for the clinic.

**Main outcome measures:**

Atrial fibrillation by 12 lead electrocardiogram (earlier study) or electrocardiogram rhythm strip (AliveCor® device) (recent study).

**Results:**

The age standardised prevalence of AF was similar between the two studies (1.6% in the 2001–2003 study and 1.8% in the 2016–2018 study, 95% confidence interval of difference −0.010, 0.014, *p* = 0.375). The prevalence in participants aged ≥65 years was 3.4% (1.0% new cases) in the recent study. Predictors of AF in the earlier study were male sex, older age and previous stroke, while in the recent study they were previous stroke and self‐reported diabetes. AliveCor® traces were successfully classified by the in‐built algorithm (91%) vs physician (100%).

**Conclusion:**

The prevalence of AF among community‐based participants in regional Victoria was similar to predominantly metropolitan‐based studies, and was unchanged over time despite increased rates of risk factors. Electrocardiogram rhythm strip investigation was successfully utilised, and particularly benefited from physician overview.

**What is already known on this subject:**

- Atrial fibrillation is an important risk factor for stroke
- Key risk factors for stroke and atrial fibrillation such as advanced age, obesity and hypertension are becoming more common and are often higher in rural areas than metropolitan areas
- The prevalence of atrial fibrillation in Australia is approximately 1–2%, but prevalence in rural areas is not well described

**What does this study add:**

- The prevalence of atrial fibrillation in this rural Victorian study was similar to other, predominantly metropolitan, Australian studies
- The prevalence was similar between two studies spaced 15 years apart despite increases in key risk factors
- The use of new AF detection technology was useful in this community‐based study, particularly with physician support

## 1 INTRODUCTION

Atrial Fibrillation (AF) is the most common cardiac arrhythmia, and an important risk factor for stroke.^1^ As AF may be asymptomatic, it can go undiagnosed until a thromboembolic or other acute event occurs. Globally, the crude prevalence of AF is increasing, in association with population ageing, and the growing prevalence of obesity and hypertension.^2^ The risk of stroke among patients with AF can be estimated by using tools such as CHADS‐2 (Congestive heart failure, Hypertension, Age [≥75 years], Diabetes Mellitus, Stroke), CHADS‐2‐VASc (CHADS‐2 with addition of Vascular disease, Age (65–74 years) and Sex (female)) or Anticoagulation and Risk Factors in Atrial Fibrillation (ATRIA).^3^

In Australia, AF prevalence or incidence has been assessed in a small number of studies, notably a modelled estimate based on the Australian population,^4^ the Busselton study of people aged 60+ conducted between 1966 and 1981,^5^ the AusDiab study^6^ and Avoid Stroke as soon as possible (ASAP) study,^7^ as reviewed by Wong.^8^ In comparison to other countries, there is a paucity of data regarding AF prevalence in Australia,^8^ particularly in rural areas.

Key risk factors for atrial fibrillation and stroke are more common in rural areas than in metropolitan areas. For example, in Australia, rural populations tend to be older,^9^ have a higher age standardised prevalence of obesity^10^ but similar rates of hypertension^11^ to metropolitan areas. Diabetes prevalence is similar in metropolitan areas and inner regional areas, although outer regional and remote areas exhibit higher diabetes prevalence.^12^ Underutilisation of anticoagulant therapy among patients with AF remains an issue in Australia and a study of 600 patients with an indication for anticoagulant therapy in rural Western Australia reported that approximately one third of patients with AF at risk of stroke or thromboembolic event had received no anticoagulation.^13^

Opportunistic, community or clinic‐based AF screening of people aged 65+ is recommended by the National Heart Foundation in Australia.^14^ Recent changes to Australian guidelines have enabled screening using medical quality electrocardiogram (ECG) rhythm strip devices in clinic and community contexts,^14^ offering an alternative to the standard, but more time consuming, expensive 12 lead ECG. Orchard and colleagues utilised ECG rhythm strip technology to estimate AF prevalence of 1.2% among patients aged 65+ in eight General Practices in rural New South Wales.^15^

A better understanding of AF prevalence, patterns of risk factors and use of anticoagulant therapy in rural Australia may assist to reduce the risk of stroke, particularly where access to specialised acute stroke care can be limited.^9^ The Crossroads studies (Crossroads‐I 2001–2003, Crossroads‐II 2016–2018) assessed health, disease and access to health services of residents of the Goulburn Valley of regional Victoria. The primary aims of this study were to describe the prevalence of atrial fibrillation and relevant risk factors among the randomly selected, community‐based participants, at two time points. A secondary aim was to assess the use of ECG rhythm strips for investigation of AF in a rural, community‐based study.

## 2 METHODS

The Crossroads I and II studies were cross‐sectional studies, and methods have been described previously.^16^, ^17^ In brief, households were randomly selected from local government lists for specific towns in regional Victoria. Written consent was obtained for all household and clinic participants. Trained research assistants attended the households and conducted the surveys face‐ to‐face. Surveys included questions about pre‐existing health conditions (including self‐reported high blood pressure, history of myocardial infarction, stroke, diabetes, heart failure, irregular heart rhythm or thyroid disease), medications, lifestyle factors (including physical exercise, alcohol consumption and smoking) and demographic details. Sufficient exercise was defined as ≥150 min per week.^18^ Lifetime risk of alcohol consumption was defined as >10 standard drinks per week.^19^ A proportion of the adults (aged 18+ years) from the household survey were randomly selected to be invited to attend a clinic to screen for a range of health conditions and obtain anthropometric measurements, pulse and blood pressure measurements. Body mass index (BMI) was calculated as weight (kg) divided by height (m) squared, before classification as under/normal weight (BMI < 25 kg/m^2^), overweight (BMI 25–29.9 kg/m^2^) or obese (≥30 kg/m^2^). Dyslipidaemia was defined as one or more of the following: total cholesterol ≥5.5 mmol/L, low‐density lipoprotein (LDL) cholesterol ≥3.5 mmol/L, high‐density lipoprotein (HDL) cholesterol <1 mmol/L, triglycerides ≥2 mmol/L.^20^ Hypercholesterolaemia was defined as total cholesterol ≥5.5 mmol/L.^21^ CHADS‐2 was calculated for participants of the Crossroads‐II study as total of history of congestive cardiac failure (1 point), hypertension (1 point), diabetes (1 point) or stroke/transient ischaemic attack (2 points) plus age ≥ 75 years (1 point).^22^ CHADS‐2 scores are then stratified into low risk (0), moderate risk (1) or high risk (≥2). CHADS‐2‐VASc was unable to be calculated due to incomplete data regarding vascular disease.

### 2.1 Differences between crossroads‐I and II

Crossroads‐I ethics approval was granted by the Goulburn Valley Ethics Committee (GCH‐3/99). Forty‐five percent of household participants were invited to the clinic. Clinic participants were screened for AF by 12 lead electrocardiogram (ECG). Participants with pacemakers were ineligible for ECG. ECG traces were interpreted by a physician (L. Bolitho) and classified according to the Minnesota coding system.^23^ Crossroads‐II was conducted using largely similar methodology in the same region of Victoria, albeit with fewer sites and participants for pragmatic reasons. Ethics was granted by the Goulburn Valley Human Ethics Research Committee (GVH20/16). Power analysis estimated that a sample size of 900 clinic participants would be required to detect differences in undiagnosed or undermanaged disease between the Crossroads‐I and II studies.^17^ One adult household participant per household was invited to the clinic. Atrial fibrillation was screened using a handheld ECG rhythm strip device (AliveCor®, Mountain View, CA, USA) connected to an iPad (Apple, Cupertino, United States). If a trace was unable to be defined as normal or AF by the inbuilt algorithm it was reviewed by a physician and classified according to the Minnesota coding system.^23^

### 2.2 Analysis

Data were imported into SPSS (SPSS Inc. USA, version 27) with the Firth logistic regression extension for R on SPSS. Continuous data are summarised as mean and standard deviation, and categorical data as frequencies and percentages. Independent groups were compared using Welch's *t*‐test as a robust test for samples with large differences in size.^24^ Dichotomous variables were compared using Chi‐squared test. Results are reported as odds ratios (OR) with 95% confidence intervals (CI). Age‐specific prevalence was calculated by the direct method, adjusting to the 2018 Australian population.^25^ AF diagnosis by the physician was used for comparing the two cohorts. As AF was a rare outcome in this study, Firth penalised logistic regression^26^ was used to assess associations between cases of AF (dependent variable) and the independent variables of age, study period, sex, self‐reported diabetes, self‐reported high blood pressure and BMI.

## 3 RESULTS

**FIGURE 1:**
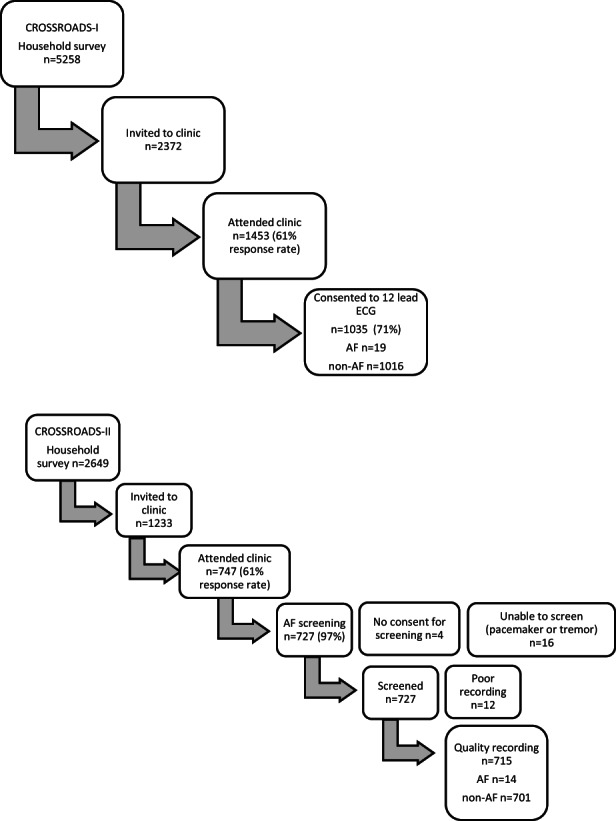
Crossroads‐I (upper panel) and crossroads‐II (lower panel) household, clinic and AF screening participants

Figure 1 shows the AF investigation uptake in the two studies. In the Crossroads‐I study, 1035 clinic participants underwent 12 lead ECG. In the Crossroads‐II study, 743 clinic participants underwent AF investigation with 16 ineligibles due to a pacemaker or tremor. The remainder (727) were screened using the handheld ECG rhythm strip (AliveCor®).

Table 1 presents the characteristics of participants in the two studies. Missing data occurred in less than 4% of cases. The average BMI of clinic participants and proportion of participants aged ≥65 years, proportion of participants with obesity, self‐reported high blood pressure or thyroid disease were significantly higher in Crossroads‐II than I.

**TABLE 1:** Demographic details of clinic participants screened for AF in each study

	Crossroads‐I	Crossroads‐II	*p*
Participants, *n*	1035	715	–
Male *n* (%)	462 (44.6)	314 (43.9)	0.807
Age (mean ± SD)	58.1 ± 13.5	59.0 ± 16.3	0.529
Age ≥65 years *n* (%)	352 (34.0)	300 (42.3)	<0.001
BMI (mean ± SD) kg/m^2^	28.1 ± 5.1	29.0 ± 6.2	0.001
Overweight *n* (%)	428 (42.2)	276 (39.9)	0.342
Obese *n* (%)	302 (29.8)	243 (35.1)	0.023
Self‐reported health conditions			
Stroke *n* (%)	32 (3.1)	26 (3.6)	0.688
High blood pressure *n* (%)	327 (31.8)	273 (38.2)	0.007
Heart failure *n* (%)	12 (1.2)	11 (1.5)	0.526
Myocardial infarction *n* (%)	21 (2.0)	26 (3.6)	0.050
Diabetes *n* (%)	98 (9.5)	86 (12.0)	0.180
Thyroid disease *n* (%)	46 (4.5)	72 (10.1)	<0.001

### 3.1 AF prevalence

The crude prevalence of AF among screened participants was 1.8% (19/1035) in the Crossroads‐I study and 2.0% (14/715) in the Crossroads‐II study. There was no significant difference in age standardised prevalence between Crossroads‐I and Crossroads‐II studies (1.6 (95% CI: 0.2–3.0%) vs 1.8 (0.1–3.4%)), 95% CI (difference): −1.3–1.4%, *p* = 0.375.

Participants with detected AF in the Crossroads I and II studies were more likely to be older, male and taking CVD medications with similar pulse rate, exercise, smoking, alcohol consumption and self‐reported thyroid disease (Table 2). Those with AF had a higher BMI and were more likely to be obese in Crossroads‐II than Crossroads‐1.

**TABLE 2:** Characteristics of clinic participants with or without detected AF

	Crossroads‐I (12 lead ECG) *n* = 1035			Crossroads‐II (ECG rhythm strip AliveCor®) *n* = 715			Crossroads‐I versus II for participants with AF detected
	AF detected (*n* = 19)	AF not detected (*n* = 1016)	*p*	AF detected (*n* = 14)	AF not detected (*n* = 714)	*p*	*p*
Male *n* (%)	15 (78.9)	447 (44.0)	0.026	9 (64.3)	305 (43.5)	0.121	0.442
Age mean ± SD	72.0 ± 13.1	57.8 ± 13.4	<0.001	69.9 ± 17.5	58.7 ± 16.1	0.029	0.696
BMI (calculated at clinic‐ kg/m^2^) mean ± SD	26.8 ± 4.4	28.1 ± 5.1	0.205	30.2 ± 3.5	29.0 ± 6.2	0.233	0.027
BMI (overweight) *n* (%)	11 (57.9)	417 (41.9)	0.169	5 (38.5)	271 (39.9)	0.612	0.296
BMI (obese) *n* (%)	2 (10.5)	300 (30.2)	0.076	8 (61.5)	235 (34.6)	0.048	0.007
Pulse mean ± SD	71.4 ± 12.5	70.1 ± 11.5	0.655	74.6 ± 14.0	71.4 ± 10.6	0.412	0.495
Pulse ≤50 BPM	1 (5.3)	29 (2.9)	0.795	0	7 (1.0)	0.707	1.000
Pulse ≥110 BPM	0	2 (0.2)	–	0	0	–	1.000
Sufficient physical exercise *n* (%)	11 (57.9)	499 (49.5)	0.469	7 (50.0)	330 (49.0)	0.939	0.733
Smoking n(%)	3 (15.8)	156 (15.4)	0.958	0	65 (9.6)	0.222	0.244
Alcohol consumption >14 drinks per week *n* (%)	0	166 (17.2)	0.965	2 (14.3)	93 (13.8)	0.958	1.000
Medical history (self‐reported)							
Stroke *n* (%)	5 (26.3)	27 (2.7)	<0.001	3 (21.4)	23 (3.3)	<0.001	1.000
Irregular heart beat *n* (%)	0	33 (3.3)	1.000	6 (66.7)	48 (6.8)	0.091	<0.001
High blood pressure *n* (%)	11 (57.9)	316 (31.3)	0.014	9 (64.3)	264 (37.7)	0.043	1.00
Myocardial infarction *n* (%)	0	21 (2.1)	1.000	0	27 (3.7)	1.000	1.000
Heart failure *n* (%)	0	12 (1.2)	1.000	0	11 (1.6)	0.637	1.000
Diabetes history *n* (%)	4 (21.1)	94 (9.3)	0.085	5 (35.7)	81 (11.6)	0.006	0.442
Thyroid condition history *n* (%)	0	46 (4.6)	0.341	0	72 (10.3)	0.206	1.000
Medications							
Medication for heart problem *n* (%)	14 (73.7)	78 (7.7)	<0.001	3 (21.4)	51 (7.3)	0.158	0.095
Medication for high blood pressure *n* (%)	10 (52.6)	292 (28.9)	0.024	6 (42.9)	174 (24.8)	0.508	0.687
Warfarin *n* (%)	Not available			1 (7.1)	1 (0.1)	0.039	NA
Other oral anti‐thrombotic agents *n* (%)	Not available			1 (7.1)	13 (1.9)	0.197	NA
Clinic blood tests							
Dyslipidaemia *n* (%)	18 (94.7)	970 (97.5)	0.452	6 (54.5)	296 (45.0)	0.527	0.016
Hypercholesterolaemia *n* (%)	4 (21.1)	402 (39.6)	0.102	2 (14.3)	216 (30.9)	0.181	1.000
CHADS‐2							
Score mean ± SD	0.5 ± 0.6	0.5 ± 0.9	0.969	1.9 ± 1.1	0.7 ± 1.0	0.001	<0.001
CHADS‐2 low (0) *n* (%)	10 (58.8)	601 (70.1)	0.213	1 (7.1)	370 (52.9)	0.001	<0.001
CHADS‐2 moderate (1) *n* (%)	6 (35.3)	161 (18.8)		4 (28.6)	187 (26.7)		
CHADS‐2 high (≥2) *n* (%)	1 (5.9)	95 (11.1)		9 (64.3)	143 (20.4)		

A majority (64.3%) of the Crossroads‐II participants with detected AF had high CHADS‐2 scores, whereas a majority of Crossroads‐I participants with detected AF had low CHADS‐2 scores (58.8%). Among participants with detected AF and high CHADS‐2 scores, only one participant in Crossroads‐II was taking warfarin and one taking another oral anticoagulant, see Table 2.

Those with self‐reported stroke were more likely to have AF (Crossroads‐I OR 12.9 (95% CI: 4.4, 38.6), Crossroads‐II OR 8.0 (95% CI 2.1, 30.8)). Similarly, those with self‐reported high blood pressure (Crossroads‐I OR 3.0 (95% CI: 1.2, 7.6) Crossroads‐II OR 3.0 (95% CI 1.0, 9.0)) or self‐reported diabetes (Crossroads‐I OR 2.6 (95% CI: 0.8, 8.0), Crossroads‐II OR 4.3 (95% CI 1.4, 13.0)) were more likely to have AF. Within the Crossroads I data set, self‐reported stroke (OR 4.70 95% CI 1.32, 14.82), age (OR 1.07 95%CI 1.02, 1.11) and male sex (OR 3.70 95% CI 1.30, 4.55) were significant predictors of AF (dependent variable) using Firth's penalised logistic regression, as per Table 3a. Similarly, within the Crossroads II data set, self‐reported stroke (OR 6.33 95% CI 1.43, 23.29) and self‐reported diabetes (OR 3.69 95% CI 1.07, 11.95) were significant predictors of AF (dependent variable) using Firth's penalised logistic regression, as per Table 3b.

**TABLE 3:** Predictors of AF, (a) crossroads‐I (firth penalised regression); (b) crossroads‐II (firth penalised regression)

	OR	95% CI of OR	*p*
(a)			
Self‐reported stroke (yes)	4.70	1.32, 14.82	0.019
Self‐reported diabetes (yes)	1.92	0.53, 5.79	0.297
Self‐reported high blood pressure (yes)	1.93	0.72, 5.32	0.191
Sex (male)	3.70	1.30, 4.55	0.013
Age (years)	1.07	1.02, 1.11	0.001
BMI	0.93	0.75, 1.04	0.224
(b)			
Self‐reported stroke (yes)	6.33	1.43, 23.29	0.018
Self‐reported diabetes (yes)	3.69	1.07, 11.95	0.040
Self‐reported high blood pressure (yes)	1.11	0.34, 3.98	0.865
Sex (male)	1.91	0.67, 5.99	0.230
Age (years)	1.04	0.99, 1.10	0.088
BMI	0.98	0.89, 1.07	0.653

### 3.2 Use of the Alive‐Core ECG rhythm strip technology

Among the 727 traces in Crossroads II, 715 (98.3%) were of high quality and of these, 639 (89.4%) were classified as normal by the in‐built algorithm, 4 (0.6%) as AF and the remainder unclassified. Upon physician review, all unclassified traces were able to be classified as normal (*n* = 701 [98.0%], with or without variation or non‐AF ECG change) or AF (*n* = 14 [2.0%]).

## 4 DISCUSSION

This study found that in this rural area the age standardised prevalence of AF was 1.6–1.8% and relatively unchanged between 2001–2003 and 2016–2018. The findings presented here address the paucity of information relating to AF prevalence and AF risk factors among community dwelling adults in rural Australia, using robust survey data. The main predictors for AF were self‐reported history of stroke, advanced age and male sex (Crossroads‐I) and self‐reported history of stroke or diabetes (Crossroads‐II), using a Firth penalised logistic regression model. Prior stroke, advanced age and diabetes are consistent independent risk factors for stroke among patients with AF reported in the literature,^27^ and thus could trigger consideration of anticoagulation for these groups of patients.

### 4.1 Prevalence

The prevalence of atrial fibrillation was similar to those reported in a study of patients aged 65+ from eight General Practices in rural New South Wales (1.2% new cases)^15^ but lower than a study of eight general practices in Sydney (3.7% AF or possible AF, new AF cases 1.1%)^28^ and 10 pharmacies also in Sydney (prevalence 6.7%, 1.5 respectively).^29^ There was no evidence of difference in AF prevalence between the two Crossroads studies 15 years apart. This was an unexpected finding given the significantly higher proportions of participants with key risk factors such as high blood pressure, obesity and advanced age in the recent Crossroads‐II study compared with Crossroads‐I. Modelling studies have predicted that ageing of the Australian population would contribute to an increase of 1.2‐fold in the incidence of AF between 2014 and 2034, with likely regional variation.^4^ There were higher proportions of participants in the more recent Crossroads‐II study with important risk factors for AF including advanced age, self‐reported high blood pressure, diabetes and obesity.

Increasing obesity prevalence has been reported in rural Australia over the past few decades^10^; however, it remains unclear whether there is a causal relationship between obesity and AF.^30^ In a meta‐analysis incorporating data from over 100 000 people, there was evidence that weight gain was associated with increased future risk of AF, but weight loss was not associated with altered risk, pointing to the importance of prevention of weight gain.^31^ However, weight loss is likely to improve cardiac risk profiles, and has been shown to reduce AF symptoms and episodes.^32^ The prevalence of diabetes (36%) among participants with AF in the Crossroads‐II study was within the range reported in a study of 9000 patients with AF stratified by BMI in the USA (18% among patients of normal weight to up to 50% among patients with obesity^33^) although prevalence of self‐reported high blood pressure (64% in Crossroads‐II) was lower (hypertension: 78% among patients of normal weight up to 90% among patients with obesity^33^). This may be due to our study detecting earlier stage and/or previously undiagnosed AF, in contrast to the patients in the Pandey study,^33^ who had been diagnosed for some time. Among AF risk factors, hypertension is thought to be the most important, due to a combination of associated risk and high prevalence.^34^

### 4.2 Value of ECG rhythm strips

ECG rhythm strips have been reported to offer a low‐cost, non‐invasive and safe solution for AF screening and linkage to treatment.^35^ ECG rhythm strip screening has been utilised in Australian General Practice^28^ and pharmacy^29^ based research studies, and have demonstrated adequate sensitivity and specificity,^36^ acceptability^28^ and cost effectiveness.^29^ Lowres and colleagues recently collated results from 19 studies using ECG rhythm strip AF screening across 14 countries and reported a pooled, age and sex adjusted detection rate of 1.4% in people aged 65 years or older.^37^The technology was useful in this community based, rural context. The algorithm was able to classify 90% of the traces as AF or normal, and each of the traces unable to be classified by the algorithm contained sufficient detail to be able to be interpreted by a physician. This agrees with Orchard and colleagues who reported that 90% of the traces in their study were able to be classified by the in‐built algorithm,^28^ but also demonstrate the need for physician overview to supplement algorithms and confirm cases of AF. Recent studies have reported that there are challenges to screening for AF in GP clinics and pharmacy settings, including a lack of time for recruitment and screening, a lack of a referral system for people with identified AF and the need for interpretation of traces unable to be classified by the algorithm.^28^, ^29^ Data from randomly selected, community based studies are important, as there is the potential for participants of GP clinic or pharmacy based studies to be more unwell than the general population.^35^ Recent changes to Australian Medicare Benefits Schedule item numbers have meant that under many circumstances, General Practitioners will not be reimbursed for interpretation of ECGs. This may reduce access to ECGs and potentially decrease identification of arrhythmia in General Practice, and has the potential to be particularly problematic in rural hospitals staffed by GPs.^38^

### 4.3 CHADS‐2, risk of stroke and anti‐thrombotic therapy

The average CHADS‐2 score (1.9 ± 1.1) and percentage of participants with a score ≥2 (64.3%) among the Crossroads‐II participants were similar to those reported among 2500 patients with AF across nine European countries (1.9 ± 1.3, 59.4%)^39^ although the percentage of CHADS‐2 scores ≥2 was lower than that reported in an Australian study of almost 20 000 Indigenous and non‐Indigenous adult patients with AF (2.0 ± 0.5, 44.1%^40^). There were few participants with AF detected who were already taking anticoagulant medication, although it is important to be cognisant of the small number of AF cases. This is unsurprising when compared to the Australian study mentioned above in which approximately 70% of patients with AF and CHADS‐2 scores of ≥2 were under‐treated with anti‐thrombotic medication.^40^ These findings suggest that ongoing education activities are required to stress the importance of anticoagulant therapy to reduce the risk of stroke in patients with AF. In addition, the use of NOACs, for which continual International Normalised Ratio (INR) monitoring is not required, may be particularly beneficial in rural areas.^14^ In addition, sustained efforts to reduce cardiovascular risk factors are important, by controlling hypertension and lipids, encouraging smoking cessation and adequate physical activity, optimising diabetes control, healthy weight, and a healthy diet rich in fibre and vegetables, and low in saturated fats.^14^, ^41^ The low proportion of cases with moderate to high CHADS‐2 scores in Crossroads‐I may suggest more undiagnosed heart disease at that time. While this cannot be substantiated, reduced access to health care, particularly primary care, was a precipitating factor for the study.^17^

### 4.4 Strengths

The large sample size and random selection of households of our study increases the generalisability of findings to similar rural areas of Australia. These findings add to the understanding of AF prevalence in rural areas. This information in turn contributes to the understanding of an important risk factor for stroke in regions where restricted access to specialist acute stroke care and higher rates of other risk factors (advanced age, obesity, chronic disease) place rural people at risk of poorer stroke outcomes.

### 4.5 Limitations

The household survey collected data regarding self‐reported health conditions and current medications. As per a cross‐sectional study design, causation was unable to be assessed. Self‐reported data such as these are associated with a degree of uncertainty and potential recall bias.^42^ Single time‐point ECG strip rhythm screening has the potential to fail to detect paroxysmal AF, which is estimated to account for one quarter of AF cases^39^ and remains an important risk factor for stroke.^1^ AF investigation methodologies differed between two study time‐points and this reduces the validity of direct comparisons of prevalence across the two‐time points. The small numbers of AF cases limit the capacity to investigate risk profiles. While the response rate to each step in the two studies was good (>60%), as a two‐step process, overall response is lower with a risk to the representativeness of the cohorts.

### 4.6 Conclusion

The prevalence of AF in this rural Victorian study of randomly selected, community participants was similar to previously reported prevalence in predominantly metropolitan studies and a recent study in rural General Practices in New South Wales. There was little evidence of change in AF prevalence despite increased rates of key risk factors. This study adds to the knowledge regarding AF prevalence and associated risk factors in rural Australia, and points to a need for sustained attention of a multitude of factors and increased use of anticoagulant therapy, in order to reduce risk of stroke. ECG rhythm strip technology was useful, particularly with physician oversight.

## AUTHOR CONTRIBUTIONS

DS, KG and L.Bourke designed the study, collected data and contributed to analysis. L.Bolitho provided ECG interpretation. KG drafted the manuscript and all authors contributed to subsequent drafts.

## CONFLICT OF INTEREST

The authors declare no conflicts of interest.

## ETHICAL APPROVAL

Crossroads‐I ethics approval was granted by the Goulburn Valley Ethics committee (GCH‐3/99). Crossroads‐II ethics approval was granted by the Goulburn Valley Human Ethics Research Committee in May 2016 (GVH20/16).
